# Assessing the community-level impact of a decade of user fee policy shifts on health facility deliveries in Kenya, 2003-2014

**DOI:** 10.1186/s12939-018-0774-4

**Published:** 2018-05-25

**Authors:** Francis Obare, Timothy Abuya, Dennis Matanda, Ben Bellows

**Affiliations:** 1 Population Council, Avenue 5, Rose Avenue, P.O. Box 17643, Nairobi, 00500 Kenya; 2 Population Council, Plot #670, No. 4 Mwaleshi Road, Olympia Park, 101010 Lusaka, Zambia

**Keywords:** User fee, Policy shifts, Maternal health service utilization, Economically disadvantaged sub-groups, Kenya

## Abstract

**Background:**

The long-term impact of user fee removal policies on health service utilization in low- and middle-income countries may vary depending on the context in which they are implemented, including whether there are policy actions to support implementation. We examined the community-level impact of a decade of user fee policy shifts on health facility delivery among poorest and rural women and compared the changes with those among the richest and urban women in Kenya using data from three rounds of nationally representative surveys.

**Methods:**

Data are from births occurring in the 5 years preceding the survey to women aged 15-49 years who were interviewed in the 2003, 2008-2009 and 2014 Kenya Demographic and Health Surveys. A total of 5949, 6079 and 20,964 births were reported in respective surveys. We conducted interrupted time series analysis predicting changes in quarterly proportions of births occurring in public and private health facilities as well as at home before and after the 2004, 2007 and 2013 user fee policy shifts in Kenya.

**Results:**

There were no statistically significant immediate changes in the proportion of births occurring in public facilities following the 2004, 2007 and 2013 user fee policy shifts among poor or rural women. There was, however, a statistically significant increase in home deliveries among all women and among those from the poorest households immediately following the 2004 policy. There was also a statistically significant increase in public facility deliveries among women from the two top quintiles, which was accompanied by a statistically decline in home deliveries immediately after the 2007 policy shift. Differences in trends in public facility deliveries between pre- and post-policy periods were not statistically significant for all sub-groups of women, indicating that even among the sub-group that experienced significant immediate increase after the 2007 policy shift, this pattern was not sustained over time.

**Conclusion:**

The findings of this paper provide empirical evidence that poorly implemented user fee removal policies benefit more well-off than poor women and in cases where there are significant immediate effects on uptake of facility delivery, this trend is not sustained over time.

**Electronic supplementary material:**

The online version of this article (10.1186/s12939-018-0774-4) contains supplementary material, which is available to authorized users.

## Background

### Introduction

The role of user fees in the health sector has dominated policy, program, and research discourse in many low- and middle-income countries (LMICs) for decades. For instance, many countries in sub-Saharan Africa introduced user fees in the health sector in the 1980s following poor economic performance, inadequate financial resources for health, declining budget allocations and international donor pressure [1]. Introducing user fees was therefore intended to generate revenue for health facilities, improve efficiency by reducing ‘frivolous’ consumption of health care services, improve quality of care, and increase coverage and utilization of services [2]. However, user fees increase out-of-pocket costs of health care and thus act as a barrier to accessing services especially among the poor thereby worsening inequities and impeding the realization of universal health care goals [3, 4]. It has also been argued that user fees generate very little revenue (between 5 and 7% of recurrent expenditure) and that their removal has little impact on facility revenue while their contribution to improving efficiency is unclear [5]. A number of sub-Saharan African countries have therefore initiated user fee removal policies for publicly-provided primary health services over the past two decades—mostly for maternal and child health services—as a means of addressing inequities in access to care [6].

Studies show that user fee removal results in improvements in health service utilization in the short-term, especially among the poor [3, 4]. However, the long-term impact of user fee removal policies is less understood. It has been argued that the gains in improving health service utilization risk being eroded over time, particularly if no alternative funding replaces the lost user fee revenue for facilities [1, 7]. In addition, the long-term impact of user fee removal on service utilization is dependent on the health systems ability to ensure adequate commodities and supplies, maintain standards of quality of care with increased client volume, and monitor delivery of services [1, 4]. Poor planning and hurried implementation may negatively affect acceptance of and compliance with the policy requiring non-payment of user fees among stakeholders involved in health service delivery, which undermines the success of user fee removal policies [1, 4, 6]. User fee removal can also greatly contribute towards achieving universal health coverage if it attracts new users who would not otherwise access services due to financial barriers rather than shifting users from one sector to another (such as from private to public since those seeking services in private facilities may have overcome some of the financial barriers). These potential limitations suggest that removing user fees requires supportive policy actions to minimize performance problems for health systems.

The foregoing discussion suggests that the long-term impact of user fee removal policies on health service utilization will vary depending on the context in which they are implemented, including whether there are policy actions in place to support implementation. In this paper, we examine changes in health service utilization between 2003 and 2014 among socio-economically disadvantaged women in Kenya following a decade of shifts in user fee policies ranging from partial to total removal of charges for maternal health services in public facilities. We specifically focus on changes in facility- and home-based deliveries among women from the bottom two household wealth quintiles and those living in rural areas, which are some of the economically disadvantaged sub-groups in terms of access to health care services that could ideally benefit the most from user fee removal policies. We compare changes among these sub-groups of women with those among women from top two household wealth quintiles and urban residents, respectively. The interest on facility deliveries was informed by the fact that user fee removal policies in Kenya (see description in the next section) were mainly aimed at reducing financial barriers to uptake of these services—given the evidence of low utilization of the services compared with professional antenatal care—with the expectation that enabling more women to deliver in health facilities under skilled care would reduce maternal and newborn morbidity and mortality in the country.

### User fee policy shifts in Kenya

Kenya has a long history of making efforts to provide health services free of charge and increase coverage. The post-colonial government made universal health care a major policy goal by abolishing user fees in 1965, 2 years after independence [8, 9]. This continued up to 1989, when the Government yielded to international pressure and introduced user fees in all levels of care and initiated other major reforms in the health sector [8, 10]. The subsequent years were characterized by suspension in 1990 and phased reintroduction of user fees from 1991 to 2003 [8, 11].

In 2004, the Ministry of Health (MOH) implemented the “10/20 policy” for maternal health services in public facilities, that removed user fees at the lowest levels of care (dispensaries and health centres) but established a registration fee of 10 shillings (approximately $ 0.1 at current exchange rate) at dispensaries and 20 shillings (approximately $ 0.2 at current exchange rate) at health centres [8]. The policy exempted children under 5 years, the poor, and those with special conditions such as malaria and tuberculosis from paying for services [8]. However, the 10/20 policy did not identify replacement funding for these public facilities and was not specific about user fees at hospitals, which are higher levels of care.

**Fig. 1:**
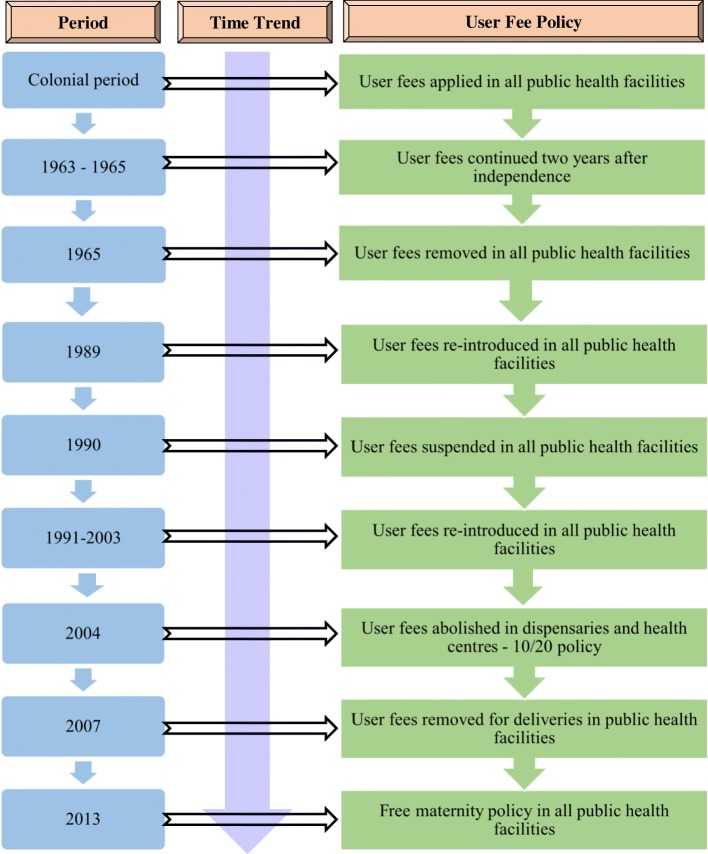
Trends in user fee policy shifts in Kenya from pre-independence period to 2013

In 2007, the 10/20 policy was removed and a policy of no user fees for deliveries in public facilities was declared. However, no alternative source of funding was offered and the reality of informal fees remained in place for many service users. In 2010, the Health Sector Services Fund (HSSF) was introduced to compensate facilities for lost revenue resulting from user fee removal and involved direct channelling of funds to bank accounts of health centres and dispensaries [8, 12, 13]. Although user fees were removed in principle, most public health facilities continued to levy charges on other components of care such as delivery supplies. Thus, following the 2013 general elections that resulted in changes in political leadership, the Government announced free maternity services in all public health facilities in June 2013 that eliminated all levies, which was the policy in place at the time of writing this paper [14]. Under the policy, facilities are reimbursed quarterly, with dispensaries and health centres receiving Kenya Shillings (KSh.) 2500 (approximately US $25), hospitals getting KSh. 5000 (approximately US $50), and referral hospitals receiving KSh. 17,500 (approximately US $175) for each delivery conducted [14]. Figure 1 summarizes the trends in user fee policy shifts in Kenya from the pre-independence era to 2013.

Available evidence shows that there has been gradual increase in revenue generated from user fees in public health facilities in the country over the years, reaching US$ 25.7 million in 2008 [8]. Out-of-pocket expenditure consistently comprised the largest share of total health care expenditure in the country over the years [8, 15]. In 2008, for instance, out-of-pocket spending on health care comprised 46% of total health care expenditure, government spending made up 39%, while the remainder came from private sources such as insurance and donors [8, 15, 16]. Health insurance coverage in the country is still low (about 10%), with heavy bias towards those in formal employment and those residing in urban areas, although recent initiatives by the National Hospital Insurance Fund (NHIF)—a mandatory national scheme for all formal sector employees—have targeted voluntary enrolment of those in informal sector [8]. The removal of user fees for services therefore implies that public health facilities have to heavily rely on government allocations for the sector based on revenue generated from taxes. In the 2008/2009 financial year, for example, such allocation accounted for only 6% of the total government budget [8].

## Methods

### Data

Data are from births occurring in the 5 years preceding the survey to women aged 15-49 years who were interviewed in the 2003, 2008-2009, and 2014 Kenya Demographic and Health Surveys (KDHS). KDHS is a nationally representative survey of women of reproductive age. A total of 8195, 8444, and 31,079 were interviewed in the 2003, 2008-2009, and 2014 surveys respectively. In all surveys, women who had ever given birth were asked detailed questions about each of the births occurring in the 5 years preceding data collection, including date and place of birth of the child. A total of 5949, 6079, and 20,964 births were reported in 2003, 2008-2009, and 2014 surveys respectively, with period of occurrence from 1998 to 2014. The sample size in the 2014 survey was substantially larger than the previous surveys because of the need to provide reliable estimates at the county level (Kenya has 47 counties). Counties were a creation of the 2010 Constitution [17]. The sample sizes for the previous surveys, on the other hand, provided reliable estimates up to provincial level—Kenya had eight provinces which ceased to exist with the promulgation of the 2010 Constitution.

### Analysis

The outcome of the analysis was quarterly trends in the proportions of births occurring in public and private health facilities as well as at home. Using information on month and year of birth of the child, we computed the total number of births occurring within each quarter of a calendar year and determined the unweighted proportion that occurred at home, public, and private health facilities during each quarter. The computation of proportions was informed by the fact that basing the analysis on absolute numbers of births would be affected by the different sample sizes; the 2014 survey had a larger sample size and higher absolute number of births. Aggregating over quarterly periods was intended to achieve reasonable numbers of births in each interval for modelling purposes.

We conducted interrupted time series analysis predicting the quarterly proportions of births occurring in public and private health facilities as well as at home before and after the 2004, 2007, and 2013 policy shifts for women from the bottom two wealth quintiles and those living in rural areas. We also provide estimates for women from the top two quintiles, those living in urban areas as well as all women aged 15-49 years. Separate models were estimated for each sub-group of women for each point of delivery. The basic model is of the following form [18]:

1 $$ {Y}_t={\beta}_0+{\beta}_1{\mathrm{T}}_t+{\beta}_2{\mathrm{X}}_t+{\beta}_3{\mathrm{X}}_t{\mathrm{T}}_t+{\varepsilon}_t $$

where *Y*_*t*_ is the outcome of interest, *β*_*0*_ is the baseline level of the outcome at the beginning of the period, *β*_*1*_ captures the trajectory of the outcome until the implementation of the policy shift, *β*_*2*_ is the change in the level of the outcome immediately after the policy shift (immediate effect of the intervention), and *β*_*3*_ is the difference in the trajectory of the outcome between pre- and post-shifts in policy (effect of the intervention over time). The covariates are defined as follows: *T*_*t*_ is the time from the start to the end of the period, *X*_*t*_ is a dummy variable coded 0 and 1 for periods before and after policy shifts respectively, *X*_*t*_*T*_*t*_ is an interaction term between time and intervention dummy, and *ε*_*t*_ is the error term [18, 19]. We further obtained the predicted post-intervention linear trends in deliveries after model estimation. The analysis was conducted using *itsa* command for time series analysis in Stata® version 14, with *prais* option to take into account auto-correlation in the time series data and *figure* option to generate graphical trends [18]. Results are presented in tabular and graphical forms.

### Ethical approval

Ethical approval for the study was obtained from the Institutional Review Board of the Population Council (Protocol 727) and AMREF Ethics and Scientific Review Committee (AMREF-ESRC P222/2016).

## Results

### Trends in maternal health service utilization in Kenya

Estimates from KDHS show that use of antenatal and delivery care services steadily increased between 2003 and 2014. The proportion of expectant women obtaining antenatal care services from a trained health care provider (doctor, nurse, or midwife) steadily increased from 88% in 2003 to 96% in 2014 while the proportion of births occurring in a health facility increased from 40 to 61% over the same period (Table 1).

**Table 1:** Trends in maternal health care utilization indicators by selected socio-demographic characteristics, Kenya 2003-2014

Characteristics	Antenatal care from a trained health care provider^a^			Facility delivery		
	2003 (%)	2008-09 (%)	2014 (%)	2003 (%)	2008-09 (%)	2014 (%)
Residence						
Urban	93.2	95.8	97.8	70.2	74.7	82.0
Rural	86.8	90.3	94.0	33.2	35.4	49.5
Household wealth quintile						
Lowest quintile	75.1	83.6	88.5	16.0	18.0	30.1
Second quintile	87.4	92.7	95.5	31.1	30.4	49.1
Middle quintile	92.4	93.2	97.1	36.5	41.6	62.3
Fourth quintile	93.0	92.7	97.4	53.2	51.4	79.9
Highest quintile	93.9	95.6	98.8	73.8	80.9	92.7
Location of service delivery						
Public facility	71.1	83.0	84.0	26.1	32.3	46.0
Private facility	27.9	16.4	16.8	14.0	10.3	15.2
Total	88.1	91.5	95.5	40.1	42.6	61.2

Note: ^a^Trained health care provider: doctor, nurse or midwife

Source: [27–29]

There were wider socio-economic disparities in facility delivery than in the use of antenatal care services offered by trained health care providers (doctors, nurses or trained midwives) over time (Table 1). For instance, the proportion of expectant women obtaining antenatal care from a trained health care provider was lower in the rural than in the urban areas by six percentage points or less across the years while the proportion of births occurring in a facility was nearly twice or more than two times higher in urban than in rural areas over the period (Table 1). Similarly, the proportion of expectant women obtaining antenatal care from a trained health care provider was between 10 and 20 percentage points lower among those from the poorest than among those from the richest households while the proportion of births occurring in a facility was more than three times higher among the richest than in the poorest households over the period (Table 1).

Most women obtained maternal health care services from public facilities across the survey years. The proportion obtaining antenatal care services from public health facilities increased from 71% in 2003 to 83% in 2008-2009 while the proportion obtaining the services from private facilities declined over the same period (Table 1). Similarly, the proportion of births occurring in public facilities steadily increased from 26% in 2003 to 46% in 2014 while the proportion occurring in private facilities declined from 14% in 2003 to 10% in 2008-2009 before reverting to the 2003 levels in 2014 (Table 1).

### Changes in public facility deliveries

There was no significant change in the proportion of births occurring in public facilities among all sub-groups of women immediately following the 2004 10/20 and 2013 free maternity policies (Table 2 and Appendix). However, there was a statistically significant positive increase in the proportion of public facility deliveries among women from the top two wealth quintiles (*p* < 0.05) but not among other sub-groups of women immediately following the 2007 policy (Table 2). In particular, there was an immediate increase of about 9% in the proportion of public facility deliveries among women from the top two wealth quintiles following the 2007 policy. The results further show that the differences between pre- and post-policy trends in public facility deliveries were not statistically significant for all sub-groups of women across all policy shifts (Table 2 and Appendix). This suggests that even for the sub-group of women that experienced statistically significant increase in public facility deliveries immediately following the 2007 policy, the trend was not sustained over time.

**Table 2:** Results from interrupted time series analysis predicting trends in public facility deliveries following 2004, 2007, and 2013 user fee policy shifts

Indicator	Bottom two quintiles		Top two quintiles		Rural women		Urban women	
	Estimate (95% CI)	*p*-value	Estimate (95% CI)	*p*-value	Estimate (95% CI)	*p*-value	Estimate (95% CI)	*p*-value
Pre-policy trend (*β*_*1*_)	0.003 (−0.000; 0.007)	0.057	0.005 (0.002; 0.009)	***0.003***	0.001 (− 0.001; 0.004)	0.340	0.005 (0.001; 0.009)	***0.033***
2004 policy								
Change in level (*β*_*2*_)	−0.059 (− 0.137; 0.019)	0.136	0.001 (− 0.080; 0.082)	0.983	0.010 (− 0.057; 0.077)	0.760	− 0.042 (− 0.143; 0.059)	0.982
Change in trend (*β*_*3*_)	0.003 (− 0.007; 0.013)	0.524	− 0.009 (− 0.020; 0.002)	0.091	− 0.000 (− 0.009; 0.008)	0.979	−0.001 (− 0.014; 0.013)	0.926
Predicted trend	0.007 (− 0.003; 0.016)	0.166	−0.004 (− 0.014; 0.006)	0.435	0.001 (− 0.007; 0.009)	0.762	0.004 (− 0.008; 0.016)	0.519
2007 policy								
Change in level (*β*_*2*_)	−0.017 (− 0.100; 0.065)	0.675	0.090 (0.004; 0.177)	***0.041***	0.017 (− 0.054; 0.088)	0.637	0.042 (− 0.066; 0.150)	0.438
Change in trend (*β*_*3*_)	−0.000 (− 0.010; 0.010)	0.970	0.008 (− 0.003; 0.019)	0.139	0.003 (− 0.005; 0.012)	0.455	− 0.004 (− 0.017; 0.010)	0.586
Predicted trend	0.006 (0.003; 0.010)	***0.000***	0.004 (0.001; 0.008)	***0.026***	0.004 (0.002; 0.007)	***0.002***	0.000 (−0.004; 0.005)	0.874
2013 policy								
Change in level (*β*_*2*_)	0.016 (− 0.085; 0.117)	0.756	−0.001 (− 0.102; 0.099)	0.977	0.019 (− 0.072; 0.111)	0.670	0.047 (− 0.077; 0.171)	0.451
Change in trend (*β*_*3*_)	−0.010 (− 0.047; 0.027)	0.589	0.017 (− 0.019; 0.053)	0.358	− 0.000 (− 0.034; 0.034)	0.988	0.019 (− 0.025; 0.063)	0.398
Predicted trend	− 0.004 (− 0.040; 0.033)	0.847	0.021 (− 0.015; 0.056)	0.249	0.004 (− 0.030; 0.038)	0.807	0.019 (− 0.025; 0.063)	0.385
Constant (*β*_*0*_)	0.110 (0.062; 0.158)	***0.000***	0.344 (0.294; 0.395)	***0.000***	0.210 (0.170; 0.249)	***0.000***	0.370 (0.306; 0.433)	***0.000***
Number of observations	64		66		65		65	

*p*-values in bold italics are statistically significant at *p* < 0.01 or *p* < 0.05

Results in Table 2 and Appendix also show that there was a statistically significant positive trend in public facility deliveries among women from the top two wealth quintiles (*p* < 0.01), those living in urban areas (*p* < 0.05), and all women (*p* < 0.05) before the 2004 10/20 policy. The proportion of births delivered in public health facilities increased by 0.5% among urban women and those from the top two wealth quintiles, and by 0.4% among all women before the 2004 policy.

**Fig. 2:**
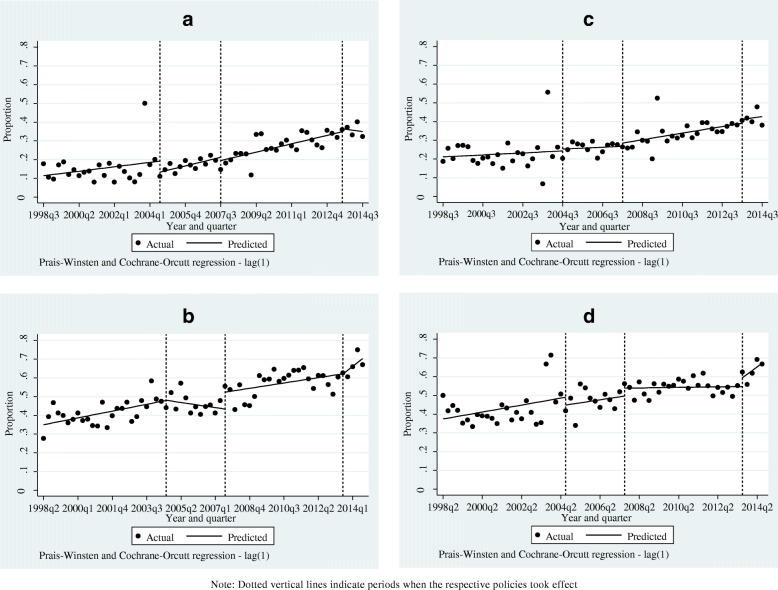
**a-d** Actual and predicted trends in public facility deliveries following 2004, 2007, and 2013 user fee policy shifts. Note: Dotted vertical lines indicate periods when the respective policies took effect

The predicted linear trends in public facility deliveries following the 2007 policy shift were positive for all sub-groups of women but only statistically significant for those from the bottom two quintiles (increase of 0.6%; *p* < 0.01), women from top two quintiles (increase of 0.4%; *p* < 0.05), rural women (increase of 0.4%; *p* < 0.01), and all women (increase of 0.4%; *p* < 0.05) (Table 2 and Appendix). These patterns are also consistent with the trends shown in Fig. 2a-d and Additional file 1.

### Changes in private facility deliveries

There were statistically significant reductions in private facility deliveries immediately following the 2004 10/20 policy among women from the bottom two quintiles (*p* < 0.01), rural (*p* < 0.01), and all women (*p* < 0.05; Table 3 and Appendix). The proportion of private facility deliveries declined by about 9, 7 and 4% every quarter among women from the bottom two quintiles, rural and all women respectively). There were, however, no statistically significant differences in trends in private facility deliveries before and after the policy shifts (2004, 2007, and 2013) among most sub-groups of women except those from the top two wealth quintiles. Among this latter group, the proportion of private facility deliveries significantly declined over time by about 3% following the 2013 policy shift (*p* < 0.05; Table 3). For those from the bottom two quintiles, rural and all women, lack of significant differences between pre- and post-policy periods indicates that the statistically significant declines in private facility deliveries that occurred immediately following the 2004 policy shift were not sustained over time.

**Table 3:** Results from interrupted time series analysis predicting trends in private facility deliveries following 2004, 2007, and 2013 user fee policy shifts

Indicator	Bottom two quintiles		Top two quintiles		Rural women		Urban women	
	Estimate (95% CI)	*p*-value	Estimate (95% CI)	*p*-value	Estimate (95% CI)	*p*-value	Estimate (95% CI)	*p*-value
Pre-policy trend (*β*_*1*_)	0.002 (−0.001; 0.004)	0.154	−0.003 (− 0.006; − 0.001)	***0.017***	0.001 (− 0.001; 0.003)	0.380	− 0.004 (− 0.008; − 0.001)	**0.030**
2004 policy								
Change in level (*β*_*2*_)	− 0.089 (− 0.145; − 0.033)	***0.002***	− 0.012 (− 0.075; 0.051)	0.699	− 0.076 (− 0.119; − 0.034)	***0.001***	0.038 (− 0.041; 0.117)	0.340
Change in trend (*β*_*3*_)	0.000 (− 0.007; 0.007)	0.935	0.007 (− 0.001; 0.015)	0.074	0.002 (− 0.003; 0.008)	0.397	0.003 (− 0.008; 0.013)	0.575
Predicted trend	0.002 (− 0.005; 0.009)	0.537	0.004 (− 0.004; 0.012)	0.294	0.003 (− 0.002; 0.008)	0.225	− 0.001 (− 0.011; 0.009)	0.836
2007 policy								
Change in level (*β*_*2*_)	−0.010 (− 0.069; 0.048)	0.742	− 0.027 (− 0.094; 0.040)	0.418	− 0.015 (− 0.061; 0.030)	0.496	− 0.022 (− 0.106; 0.063)	0.611
Change in trend (*β*_*3*_)	− 0.002 (− 0.009; 0.005)	0.631	−0.002 (− 0.010; 0.006)	0.674	− 0.003 (− 0.008; 0.003)	0.345	0.000 (− 0.010; 0.011)	0.960
Predicted trend	0.000 (− 0.002; 0.003)	0.742	0.002 (− 0.000; 0.005)	0.088	0.001 (− 0.001; 0.002)	0.511	− 0.001 (− 0.004; 0.003)	0.675
2013 policy								
Change in level (*β*_*2*_)	−0.005 (− 0.079; 0.070)	0.903	0.025 (− 0.056; 0.105)	0.543	− 0.007 (− 0.064; 0.050)	0.800	0.016 (− 0.080; 0.113)	0.733
Change in trend (*β*_*3*_)	− 0.001 (− 0.029; 0.026)	0.915	− 0.030 (− 0.059; − 0.001)	***0.042***	− 0.005 (− 0.026; 0.016)	0.619	− 0.015 (− 0.049; 0.019)	0.389
Predicted trend	− 0.001 (− 0.028; 0.026)	0.938	−0.028 (− 0.057; 0.001)	0.059	−0.005 (− 0.026; 0.016)	0.659	−0.016 (− 0.050; 0.018)	0.361
Constant (*β*_*0*_)	0.068 (0.034; 0.102)	***0.000***	0.283 (0.245; 0.322)	***0.000***	0.099 (0.073; 0.125)	***0.000***	0.307 (0.256; 0.358)	**0.000**
Number of observations	64		65		64		65	

*p*-values in bold italics are statistically significant at *p* < 0.01 or *p* < 0.05

**Fig. 3:**
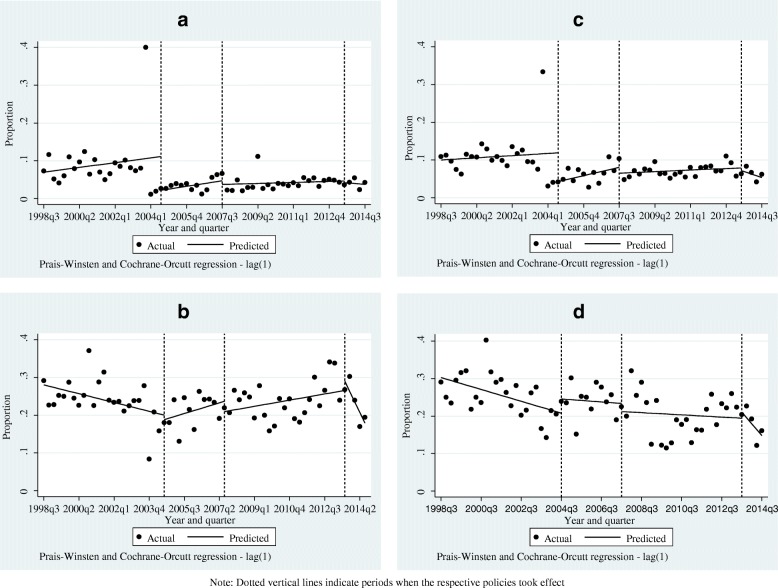
**a-d** Actual and predicted trends in private facility deliveries following 2004, 2007, and 2013 user fee policy shifts. Note: Dotted vertical lines indicate periods when the respective policies took effect

The pre-2004 policy trends in private facility deliveries among women from the top two quintiles and those in urban areas show statistically significant declines of about 0.3 and 0.4%, respectively, (*p* < 0.05 in each case). The directions of the predicted trends in private facilities differed by sub-groups of women and policy shift although in all cases, the trends were not statistically significant (Table 3). These patterns are also reflected in the trends shown in Fig. 3a-d and Additional file 1.

### Changes in home deliveries

There was statistically significant positive change in home-based deliveries among women from the bottom two quintiles (*p* < 0.01) and among all women (*p* < 0.05) immediately following the 2004 the 10/20 policy (Table 4 and Appendix). The proportion of deliveries occurring at home increased by about 12 and 9% among these sub-groups of women, respectively, following the 2004 policy shift. In contrast, the 2007 and 2013 policy shifts did not have significant immediate effect on home-based deliveries among most sub-groups of women except for those from the top two wealth quintiles, who experienced a statistically significant decline of about 6% immediately following the 2007 policy shift (*p* < 0.05). There was also a statistically significant decline (of about 0.7%) in the proportion of home deliveries among this sub-group of women over time (*p* < 0.05) in the period following the 2007 policy shift (Table 4).

**Table 4:** Results from interrupted time series analysis predicting trends in home-based facility deliveries following 2004, 2007, and 2013 user fee policy shifts

Indicator	Bottom two quintiles		Top two quintiles		Rural women		Urban women	
	Estimate (95% CI)	*p*-value	Estimate (95% CI)	*p*-value	Estimate (95% CI)	*p*-value	Estimate (95% CI)	*p*-value
Pre-policy trend (*β*_*1*_)	−0.003 (− 0.006; 0.001)	0.111	− 0.001 (− 0.003; 0.002)	0.585	− 0.001 (− 0.004; 0.002)	0.567	− 0.002 (− 0.006; 0.002)	0.287
2004 policy								
Change in level (*β*_*2*_)	0.111 (0.028; 0.194)	***0.009***	−0.004 (− 0.054; 0.045)	0.865	0.048 (− 0.025; 0.122)	0.193	0.017 (− 0.066; 0.100)	0.683
Change in trend (*β*_*3*_)	−0.006 (− 0.017; 0.004)	0.233	0.001 (− 0.006; 0.007)	0.821	− 0.004 (− 0.014; 0.005)	0.372	− 0.001 (− 0.012; 0.010)	0.898
Predicted trend	− 0.009 (− 0.019; 0.001)	0.075	0.000 (− 0.006; 0.006)	0.962	− 0.005 (− 0.014; 0.004)	0.256	− 0.003 (− 0.013; 0.008)	0.592
2007 policy								
Change in level (*β*_*2*_)	0.031 (− 0.058; 0.120)	0.490	−0.064 (− 0.117; − 0.011)	***0.019***	0.001 (− 0.077; 0.079)	0.977	− 0.027 (− 0.115; 0.061)	0.547
Change in trend (*β*_*3*_)	0.003 (− 0.008; 0.013)	0.641	−0.007 (− 0.013; − 0.001)	***0.037***	0.000 (− 0.009; 0.010)	0.947	0.003 (− 0.008; 0.014)	0.604
Predicted trend	− 0.007 (− 0.010; − 0.003)	***0.001***	− 0.007 (− 0.000; − 0.005)	***0.000***	− 0.005 (− 0.008; − 0.002)	***0.003***	−0.000 (− 0.004; 0.004)	0.938
2013 policy								
Change in level (*β*_*2*_)	−0.016 (− 0.122; 0.090)	0.760	− 0.016 (− 0.082; 0.051)	0.640	−0.016 (− 0.113; 0.081)	0.743	−0.062 (− 0.161; 0.036)	0.211
Change in trend (*β*_*3*_)	0.011 (−0.027; 0.049)	0.572	0.010 (−0.014; 0.035)	0.393	0.006 (−0.030; 0.041)	0.750	−0.005 (− 0.040; 0.030)	0.775
Predicted trend	0.004 (−0.034; 0.042)	0.829	0.004 (−0.020; 0.028)	0.755	−0.001 (− 0.034; 0.036)	0.962	−0.005 (− 0.040; 0.030)	0.779
Constant (*β*_*0*_)	0.799 (0.748; 0.849)	***0.000***	0.344 (0.314; 0.374)	***0.000***	0.673 (0.628; 0.717)	***0.000***	0.338 (0.283; 0.392)	***0.000***
Number of observations	66		65		65		65	

*p*-values in bold italics are statistically significant at *p* < 0.01 or *p* < 0.05

**Fig. 4:**
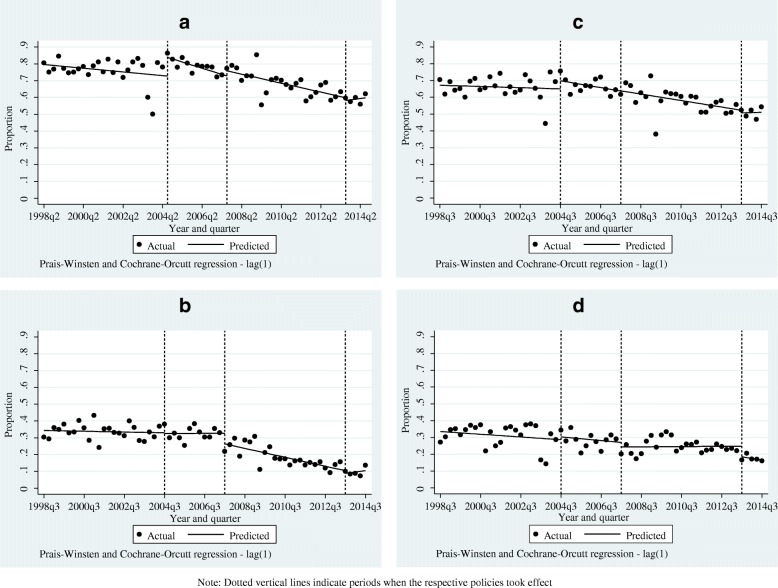
**a-d** Actual and predicted trends in home-based deliveries following 2004, 2007, and 2013 user fee policy shifts. Note: Dotted vertical lines indicate periods when the respective policies took effect

Results in Table 4 further show that there was non-significant negative trend in home deliveries in the period before the 2004 policy shift among all sub-groups of women considered. In addition, the predicted linear trends in home deliveries following the various policy shifts were negative among all sub-groups of women. The predicted negative trends were, however, statistically significant for women from the bottom two quintiles (decline of 0.7%; *p* < 0.01), those from the top two quintiles (decline of 0.7%; *p* < 0.01), rural women (decline of 0.5%; *p* < 0.01), and all women (decline 0.5%; *p* < 0.05; Table 4) after the 2007 policy shift. These patterns are also consistent with the trends shown in Fig. 4a-d and Additional file 1.

## Discussion

We examined the community-level impact of a decade of user fee policy shifts on health facility deliveries among economically disadvantaged (poorest and rural) women in Kenya using data from nationally representative surveys and compared the changes with those among richest and urban women. One finding is that there were no statistically significant immediate changes in the proportion of births occurring in public facilities following the 2004, 2007 and 2013 user fee policy shifts among economically disadvantaged women considered. There was, however, a statistically significant increase in public facility deliveries among women from the two top quintiles, which was accompanied by a statistically decline in home deliveries immediately after the 2007 policy shift. Differences in trends between pre- and post-policy periods were not statistically significant for all sub-groups of women, indicating that even among women from the top two wealth quintiles who experienced significant immediate increase in public facility deliveries after the 2007 policy shift, this trend was not sustained over time.

The finding on changes in public facility deliveries among various sub-groups of women following the various policy shifts is consistent with some of the existing evidence that removal of user fees alone might not be sufficient to increase utilization of services among economically disadvantaged women unless accompanied with interventions to address other barriers to accessing health care such as distance to care, inadequate staff, lack of commodities and supplies, poor quality services and cultural barriers [4, 7, 20]. Changes among poor and economically well-off women following the 2007 policy shift also indicate that poorly implemented user fee removal policies may benefit better-off more than economically disadvantaged population sub-groups. The finding is further consistent with the evidence which shows that even if cost of health care services is highly subsidized for economically disadvantaged groups such as through the use of vouchers, segments of intended beneficiaries still do not seek care because of some of these barriers [21, 22].

The results of the paper further show that although there was no immediate significant effect of the 2007 policy on public facility deliveries among poorest, rural and all women, the predicted rate of increase in such deliveries was statistically significant. This was accompanied by statistically significant changes in the predicted rate of decline in home deliveries among these sub-groups of women following the policy. The finding suggests that at the population level, the 2007 policy might have accelerated but not dramatically altered the trends in public facility deliveries among poorest, rural and all women, with concomitant significant shifts in the rate of decline in home deliveries. This is intuitive as births occurring in public facilities were already on an upward trajectory while those occurring at home were on a downward trajectory prior to 2007; hence removal of user fees accelerated rather than dramatically altered the trends. The statistically insignificant rate of change after the 2013 user fee removal policy could, on the other hand, be partly due to the fact that the 2014 survey was conducted a few months after the policy came into effect when no meaningful change could be detected.

There were statistically significant declines in private facility deliveries among poorest and rural women as well as among all women immediately following the 2004 introduction of the 10/20 policy. An intriguing aspect is that despite these shifts in private facility deliveries immediately following the policy, there was no immediate concomitant increase in public facility deliveries among these sub-groups of women. Rather, there was a statistically significant increase in home deliveries among all women and among those from the poorest households immediately following the policy. This suggests that even modest user fee charges drive the poorest women from delivering in a health facility. In addition, there is evidence that implementation of the policy was affected by poor design, unclear guidelines and negative attitudes of health care staff, and that although there was an increase in public facility deliveries following its implementation, this was not sustained [1, 8]. Given that the 2004 10/20 policy did not concern deliveries in private health facilities, the shift from such facilities immediately following its coming into effect might have been influenced by other factors not captured by the data such as local misunderstanding of its implementation.

The detriments of charging user fees especially with respect to perpetuating inequities in access to health care services and the challenges associated with implementing user fee removal policies in many LMICs have led to arguments for risk-pooling initiatives such as through insurance [8, 23]. However, in Kenya as in many LMICs, only a minority of the population mainly comprising formal sector employees have health insurance cover [8]. As previously noted, the national health insurance scheme—NHIF—is mandatory for all formal sector employees and voluntary for informal sector workers. Private sector employers can also purchase private health insurance for their employees in addition to NHIF. A key challenge for the country remains how to expand health insurance coverage to the majority of, if not all, Kenyans. Efforts to introduce comprehensive health insurance coverage for all Kenyans through a national social health insurance scheme in 2004/2005 were unsuccessful due to technical and political reasons [24, 25]. Informal sector workers face various barriers to enrolment in NHIF including unclear registration requirements and processes; high premium levels that are out of reach for most of them; and unclear mechanisms for contribution [26]. Free maternity services that came into effect in 2013 have been transferred to NHIF as part of managed care but mainly for public facility deliveries while mechanisms for covering deliveries in private for- and non-profit facilities are yet to be formulated. These developments indicate that formulating an appropriate financing mechanism for achieving universal health coverage remains a major challenge for the country.

The findings of the paper might be influenced by a few limitations. First, KDHS data are cross-sectional and information on births is obtained retrospectively. Births occurring further back from the date of interview may thus be subject to recall bias. Second, some nuances might have been lost in the process of aggregating information on births on a quarterly basis. However, the analytical approach used (interrupted time series) was necessitated by the need to examine trajectories of births and whether the policy shifts contributed to significant changes in such trajectories, and such approach was not possible with individual-level data. Third, use of secondary data limited the ability to distinguish between supply- and demand-side effects of user fee policy shifts. In particular, it was not possible to determine whether changes in facility delivery were driven by user or provider behaviour, or both, in response to user fee removal. In addition, the data do not permit examining how user fees applied to the different levels of care (hospitals, dispensaries and health centres) because of the small number of deliveries occurring in lower levels of care (dispensaries and health centres) resulting in many time points with empty cells for these levels, which reduces the confidence in the estimates. The effect of removing user fees on uptake of public sector deliveries may also be influenced by household decision-making process regarding where to deliver, which may be made long before the date of delivery, although this is mostly common among those with health insurance coverage.

## Conclusion

The findings provide empirical evidence that poorly implemented user fee removal policies benefit more well-off than poor women and in cases where there are significant immediate effects on uptake of facility delivery, this trend is not sustained over time. In addition, the findings suggest that in contexts where facility deliveries are already on an upward trajectory, user fee removal initiatives accelerate the trends rather than dramatically altering them. In order to achieve universal health coverage, there is need for such initiatives to be accompanied by policy and programmatic actions to address other barriers to accessing health services, especially among economically disadvantaged segments of the population.

## Additional file

Additional file 1: Actual and predicted trends in public facility, private facility and home-based deliveries among all women following 2004, 2007, and 2013 user fee policy shifts. Note: Dotted vertical lines indicate periods when the respective policies took effect. (PDF 486 kb)

## Appendix

**Table 5:** Results from interrupted time series analysis predicting trends in public, private and home-based facility deliveries among all women following 2004, 2007, and 2013 user fee policy shifts

Indicator	Public facility deliveries		Private facility deliveries		Home-based deliveries	
	Estimate (95% CI)	*p*-value	Estimate (95% CI)	*p*-value	Estimate (95% CI)	*p*-value
Pre-policy trend (*β*_*1*_)	0.004 (0.001; 0.007)	***0.019***	−0.001 (−0.002; 0.001)	0.175	−0.003 (− 0.006; 0.000)	0.072
2004 policy						
Change in level (*β*_*2*_)	−0.039 (− 0.117; 0.039)	0.321	− 0.040 (− 0.081; − 0.000)	***0.050***	0.082 (0.000; 0.164)	***0.050***
Change in trend (*β*_*3*_)	− 0.001 (− 0.011; 0.009)	0.844	0.004 (− 0.002; 0.009)	0.161	−0.002 (− 0.013; 0.008)	0.642
Predicted trend	0.003 (−0.007; 0.012)	0.554	0.003 (−0.002; 0.007)	0.313	−0.006 (− 0.016; 0.005)	0.276
2007 policy						
Change in level (*β*_*2*_)	0.023 (−0.060; 0.107)	0.582	−0.013 (− 0.056; 0.029)	0.535	− 0.011 (− 0.099; 0.077)	0.809
Change in trend (*β*_*3*_)	0.001 (− 0.009; 0.011)	0.806	− 0.002 (− 0.007; 0.003)	0.475	0.001 (− 0.010; 0.012)	0.866
Predicted trend	0.004 (0.001; 0.008)	***0.018***	0.001 (−0.001; 0.002)	0.462	−0.005 (− 0.008; − 0.001)	***0.011***
2013 policy						
Change in level (*β*_*2*_)	0.028 (−0.072; 0.129)	0.578	0.003 (−0.050; 0.050)	0.908	−0.031 (− 0.137; 0.076)	0.568
Change in trend (*β*_*3*_)	−0.002 (− 0.038; 0.034)	0.924	− 0.009 (− 0.023; 0.005)	0.187	0.012 (− 0.027; 0.050)	0.546
Predicted trend	0.002 (− 0.034; 0.038)	0.896	− 0.012 (− 0.031; 0.008)	0.208	0.007 (− 0.031; 0.045)	0.715
Constant (*β*_*0*_)	0.233 (0.186; 0.280)	***0.000***	0.158 (0.134; 0.183)	***0.000***	0.609 (0.559; 0.659)	***0.000***
Number of observations	66		65		66	

*p*-values in bold italics are statistically significant at *p* < 0.01 or *p* < 0.05

## Abbreviations

HSSF: Health Sector Services Fund
KDHS: Kenya Demographic and Health Survey
LMICs: Low- and Middle-Income Countries
MOH: Ministry of Health
NHIF: National Hospital Insurance Fund
