# Supplementary material for: Assessing the community-level impact of a decade of user fee policy shifts on health facility deliveries in Kenya, 2003-2014
Source: Int J Equity Health. 2018 May 25;17:65. doi: 10.1186/s12939-018-0774-4 (PMC5970478; doi:10.1186/s12939-018-0774-4)
Supplement: Supplementary file 1 — Actual and predicted trends in public facility, private facility and home-based deliveries among all women following 2004, 2007, and 2013 user fee policy shifts. Note: Dotted vertical lines indicate periods when the respective policies took effect. (PDF 486 kb) [file 12939_2018_774_MOESM1_ESM.pdf]

Additional file 1 Actual and predicted trends in public facility, private facility and home-based deliveries among all women following 2004, 2007, and 2013 user fee policy shifts

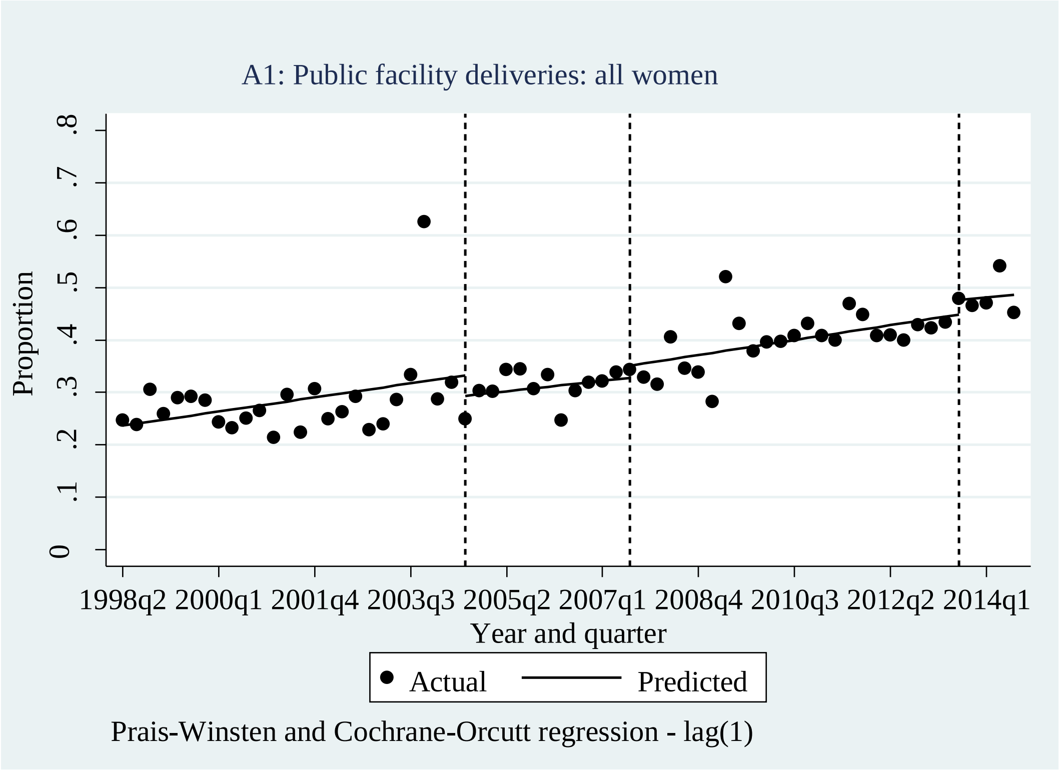

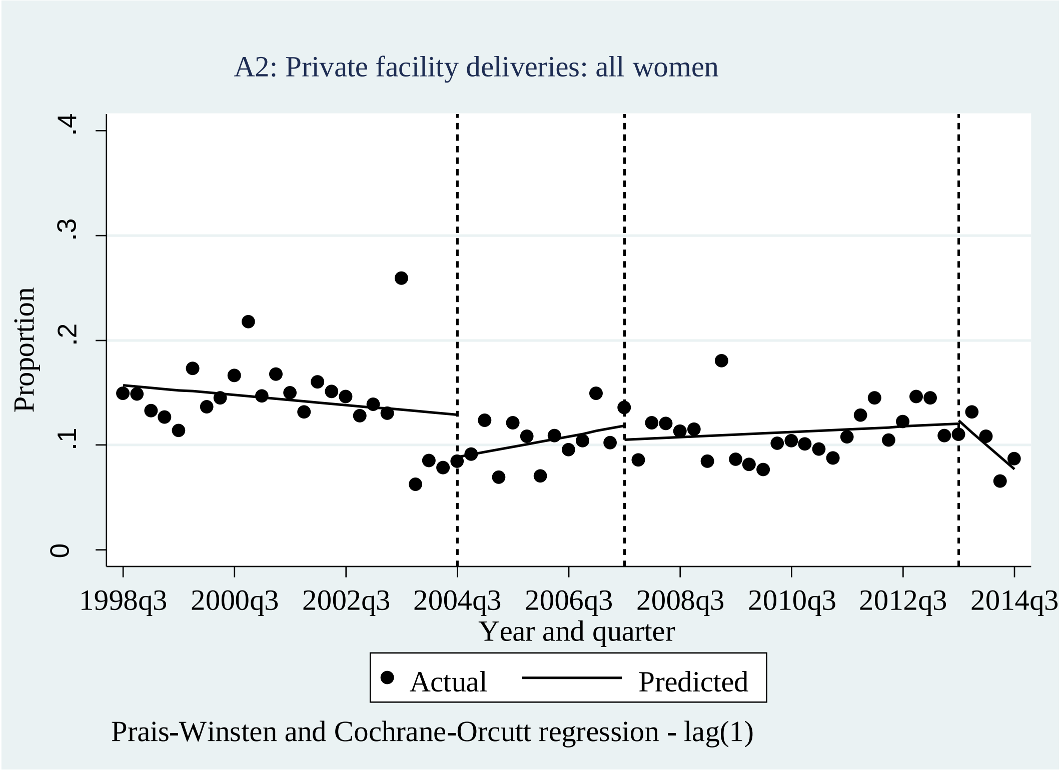

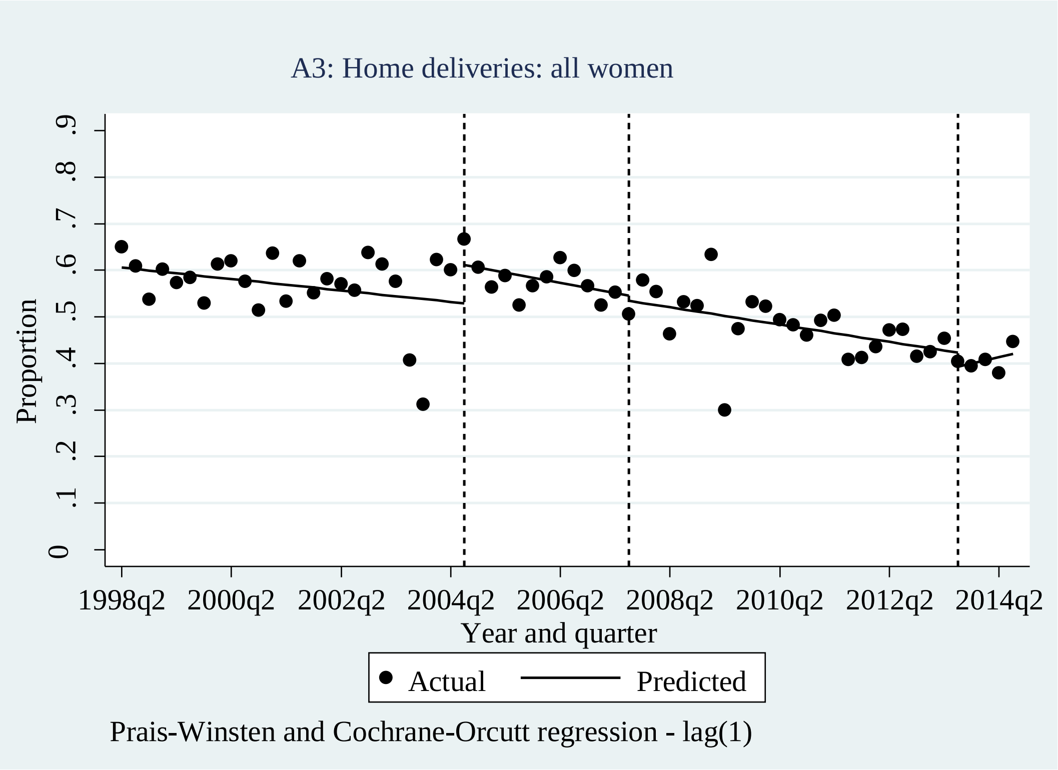
A3: Home deliveries: all women
Proportion
0
.1
.2
.3
.4
.5
.6
.7
.8
.9
1998q2
2000q2
2002q2
2004q2
2006q2
2008q2
2010q2
2012q2
2014q2
Year and quarter
Actual
Predicted
Prais-Winsten and Cochrane-Orcutt regression - lag(1)
